# Compromise, coercion, and delay: best interests decision-making in mediation of paediatric medical treatment disputes

**DOI:** 10.1093/medlaw/fwaf041

**Published:** 2025-11-27

**Authors:** Jaime Lindsey, Gillian Francis

**Affiliations:** Faculty of Law, University of Oxford, Oxford, OX1 3UL, United Kingdom; Future Pupil Barrister, Drystone Chambers, London, United Kingdom

**Keywords:** best interests, compromise, conflict, mediation, medical treatment, paediatric disputes

## Abstract

This article explores the role of mediation in best interests disputes concerning the provision of health and care to children. These cases concern disagreements between healthcare professionals and family members about which treatment should be provided. Mediation, a form of non-judicial resolution, has been argued for as an alternative and more supportive way of approaching these challenging, and often emotionally charged, disagreements. However, mediation is a confidential process, and this can lead to a lack of reliable data. Drawing on data from an empirical project, which looked at the role of mediation in medical treatment disputes, we highlight mediation’s role in securing the best interests of the child. We explore three themes related to best interests: compromise, delay, and coercion. We argue that there is limited evidence from our research of compromise or coercion through the use of mediation, albeit there is some evidence of health professionals feeling pressured into participating in mediation. However, there is evidence that mediation can cause delay in resolution. We suggest that whether this undermines the child’s best interests will depend upon the interpretation, noting that there is value in seeking agreement but the interests of the child need to be at the forefront.

## I. INTRODUCTION

This article explores the role of mediation in best interests disputes concerning the provision of health and care to children. Mediation is a conflict resolution process involving an independent third-party mediator who facilitates an attempt at resolution. It has been suggested as an alternative way of approaching emotionally charged disagreements about the provision of health and care to adults and children, including by the parents involved in high-profile cases.^1^ However, mediation is a confidential process, and this can make it difficult to gain reliable data to interrogate whether it is suitable for best interests disputes. Here, we publish data from an empirical project that looked at the role of mediation in medical treatment disputes.

One of the most high-profile cases concerning a dispute about the healthcare of a child concerned Charlie Gard, a baby who suffered from a rare and progressive genetic disease. The healthcare professionals (HCPs) and Charlie’s parents were in disagreement about the way forward. The HCPs considered that he was beyond hope of any meaningful recovery, yet Charlie’s parents disagreed with that assessment of his level of awareness, believing that alternative treatment provided him with some hope of recovery and invoking global media support for their cause. The Court agreed with the hospital trust that Charlie should only be provided with palliative care and that the treatment desired by his parents was not in Charlie’s best interests. Treatment was subsequently withdrawn, and Charlie died.^2^ The level of conflict between the family and hospital was striking, and the media attention which the case garnered also fed into the perception that an alternative way of resolving disputes such as these might be necessary, both to better support the parents and protect the HCPs from such intense scrutiny. There has also been some discussion of mediation in case law; for example, Mr Justice Francis encouraged the parties to mediate in Charlie Gard’s case.^3^

These disputes usually arise either because there is a disagreement between parent and child or, more often, a disagreement between HCPs and the child’s parents. There appears to be an increase in cases reaching the courts where parents and HCPs are in conflict about the child’s best interests.^4^ Of course, this may not be down to actual increases in conflict; there may simply be more judgements published and greater media focus on high-profile cases. Case law analysis confirms that litigation is more likely to result in an outcome agreeing with the public body rather than the family,^5^ suggesting that the best interests question is more likely to be interpreted by the courts in accordance with the public body’s view of that issue. The reasons for this are contentious but may not necessarily be due to judicial deference to the medical profession; case selection on the part of the public body, where they only pursue cases through litigation that they think they are likely to ‘win’, may also contribute to this finding. Whatever the reasons for this, it is right to say that litigation may not benefit family members seeking support for their decision-making rights in relation to their child. On the other hand, there may be a benefit to family members of having a decision taken out of their hands, particularly when withdrawal of treatment is the issue in dispute.^6^

The guiding principle where there is a disagreement about medical treatment is what is in the child’s best interests, which is not limited only to the analysis of clinical factors.^7^ The literature engaging with this topic covers issues including the causes of conflict, the meaning of best interests, the arguments for and against a change to a significant harm threshold, and the futility of treatment.^8^ There is disagreement about the meaning of the best interests concept and the extent to which it is subjective or guided by clear legal principle.^9^ However, very little of this literature engages with mediation and the ways in which it may support or undermine families in their attempts to resolve disputes with HCPs about their children.^10^ The literature that does engage with mediation suggests that either it may not be appropriate in cases where there is high conflict or considers only the theoretical and conceptual potential of mediation.^11^ With that background in mind, might mediation provide an alternative way of resolving best interests disputes between HCPs and parents in a way that better preserves relationships? To answer this, first, we outline the context of mediation in best interests disputes. Then we analyse data from our interviews, focusing on the question of whether the child’s best interests can be secured through mediation. We focus on three issues: compromise, delay, and coercion. Overall, we argue that mediation has a place in resolving paediatric disagreements. However, its potential for delay and the process coercion that some HCPs described experiencing suggest its use should be carefully considered in each case, rather than being the default position in all cases of unresolved conflict.

## II. MEDIATION IN BEST INTERESTS DISPUTES

There are different types of mediation but the focus here is on facilitative mediation, as that is the type most practised in family justice and the type that was predominantly described by our interview participants.^12^ In facilitative mediation, mediators ‘exert low control over the content of the mediation but high control over the process’.^13^ In other words, the mediator is the process expert but leaves the question of substantive justice to the parties and is non-directive about the outcome.^14^ In the context of medical treatment disputes concerning seriously unwell children, this notion can be problematic. It raises the question of who oversees the protection of the child’s best interests, the guiding legal principle in matters concerning the welfare of the child. Moreover, in cases where older children might be involved, the mediator may need to take a more active role to engage them in the process and ensure their wishes are not excluded.^15^ Our data suggest that parties are acutely alive to the best interests question and notions of mediated compromise are not borne out.

Mediators have different styles but facilitative mediation will typically involve some pre-meetings between individual parties and the mediator to help identify issues and concerns, but also for the mediator to help the parties engage with mediation with open minds rather than entrenched positions. The mediator’s role is to facilitate discussion between the parties to try and reach an agreed way forward. This may not be a complete resolution of the entire dispute but may include agreement on certain aspects (often referred to as narrowing the issues in legal terms), improved understanding of the other party’s position, and improved communication.^16^ The latter two benefits can feed into future resolution attempts, and, in cases where an ongoing relationship is required, which is often the case in medical disputes, improved understanding and communication can be hugely important. While we know that the majority of decisions about a child’s medical treatment are taken by parents in agreement with HCPs, in those small number of cases where conflict does become more severe, mediation can have benefits for family members and HCPs alike.^17^

Other benefits of mediation that may arise include improved participant wellbeing, a less adversarial and more collaborative process, greater flexibility,^18^ and improved working relationships.^19^ Mediation can also be more participatory and encourage voluntary engagement with the process of resolution.^20^ While many of these benefits are well established, more evidence is needed to show their relevance in medical treatment disputes specifically and how they might work for families.^21^ There are, however, risks in mediating medical treatment disputes, including that parties may reach agreements which are not in the child’s best interests, that family members may feel coerced into agreement with HCPs, and that the mediation process can be coercive in ways that mean it goes beyond the confines of the original dispute, sometimes described in the literature as ‘mission drift’.^22^

Despite mediation becoming embedded in civil justice^23^ and even in other areas of law, including in family law and clinical negligence,^24^ it is not yet well embedded in medical treatment disputes concerning children in the family courts. This may be because of the various concerns about mediation and the paucity of evidence on its use in medical treatment disputes. In this article, we solely engage with the best interests question and explore it through the data collected through our empirical project to argue that mediation can secure the best interests of the child, understood through the prism of how the family courts have approached the best interests question. That is not to suggest that mediation is appropriate in all cases nor do we address here some of the other challenges of mediating medical treatment disputes beyond the best interests question.^25^

## III. METHODS

We conducted 30 semi-structured interviews with participants who had experience of mediation and best interests disputes concerning adults and children. These data were collected as part of a project which also included three participant observations of mediation, although those observations are not considered in this article as no observations included children’s cases. Seventeen participants had experience of children’s medical treatment disputes, and 18 had experience of adult health and care disputes.^26^ Most of the participants with children’s experience were (primary roles) HCPs (*n* = 7), mediators (*n* = 3), lawyers (*n* = 3), chaplains (*n* = 2), and family members or supporters (*n* = 2). Some participants had secondary roles too, for example one HCP was also a mediator (Yasmin), one lawyer (Nadine) was also a mediator, one mediator was also a lawyer (Ed), one mediator was an HCP (Abigail), and one family supporter was a lawyer (Laura). All of the lawyers interviewed were involved in mediation either representing one party (Rachel represented the child’s interests, Nadine had experience of representing a range of different participants, and Oscar’s primary experience was representing public bodies). The two chaplains (Francesca and Marcus) interviewed took part in their role as supporters for families. All references to names are pseudonyms, and data have been anonymized to prevent participant identification.

Participants were recruited using snowball and purposive sampling, drawing on our existing networks of contacts. We circulated requests among participants in mediations that we knew were taking place, via social media channels, and asked participants and interested stakeholders to distribute our request. The project was also listed in the National Institute for Health Research Portal. Ethical approval was received from the Health Research Authority, and informed consent was obtained from all participants before proceeding with interviews. Ethical challenges included that mediation is confidential and therefore participants were careful about the level of information disclosed to us, focusing on their own experiences and reflections on mediation. This also means that we have very little quantitative baseline information regarding mediation to inform our findings. We do not know how many mediations take place, how often, or how successful they are.^27^ This research used a qualitative methodology to explore participant experiences of mediation and so is unable to address this question of the quantitative success of mediation, although we have included some evidence in the section below regarding mediation outcomes shared with us.

Other challenges included participant recruitment; we only interviewed one family member, a mother, whose story is explained in more detail below, as it is an important case study. We also interviewed an individual we are describing as a family supporter, as their role primarily involves supporting families through disputes of this nature. Challenges with participant recruitment were something we anticipated in advance of commencing the research.^28^ Predominantly, we experienced difficulty in identifying and contacting family members who had been directly involved in mediation, given that it is an extremely small pool of individuals who have any experience of medical mediation. Our primary recruitment contacts were mediators and lawyers, and while we understand they shared our participant request with family members, they were unable to put us in direct contact without consent. We had some success in discussions with support organizations, although this did not result in any interviews with family members, sometimes due to concerns about how much individuals were allowed to share about mediation, a lack of direct experience with mediation, or sensitivities around the subject matter and participants’ own emotional journeys in what are difficult cases. Weaknesses of the data therefore include: that we only interviewed one family member and therefore our insights are predominantly from HCPs and mediators; some HCP and legal participants misunderstood the concept of mediation (for example, they believed they had taken part in mediation but when pressed their explanation of the process was not independent mediation), and, finally, that the overall numbers of participants are relatively small. However, these numbers are compatible with the aims of qualitative research, in particular, to gain insights based on individual participant experience.

## IV. Compromise, delay, coercion, and best interests

The research underpinning this article explored the extent to which mediation can and should be used to achieve to achieve therapeutic justice in medical treatment disputes.^29^ Analysis of therapeutic justice and mediation is published elsewhere,^30^ but it is important to note here that even if mediation has therapeutic benefits for the parties, including family members, those benefits are unlikely to be sufficient if the patient’s best interests are not secured. Therefore we have analysed the data thematically for this article to draw insights into participants’ perspectives on the best interests question. This was explored through the interviews by asking participants questions such as ‘did you reach any agreements at the mediation, if so what, if not, why not?’, ‘were there any aspects of the agreement reached that surprised you?’ and ‘do you think the agreement reached was in the patient’s interests?/Do you think the agreement was fair?’. Drawing on these questions, as well as the wider narratives that participants shared with us, we have identified three themes of compromise, delay, and coercion, which are discussed to consider the ways in which mediation can secure best interests.

Before turning to that analysis, we first set out some of the outcomes that were shared with us to provide an overarching understanding of what can happen in mediation, which then informs the subsequent analysis. In mediations for disputes about children, which is the focus of this article, there tended to be four different types of outcomes at mediation.^31^ First, and most frequently in what was described to us, was no agreement at the mediation and the dispute continued (in some instances we were told specifically that the case proceeded to litigation but not always). Secondly, there were agreements made which were in accordance with the HCPs’ view of best interests (which tended to be agreements to be withdraw treatment albeit not exclusively). In four interviews we were explicitly told that there were agreements to withdraw treatment in agreement with the HCPs (Kai, Lola, Abigail, Marucs). Thirdly, there were sometimes agreements where certain aspects of the dispute were resolved such as agreements about timing or locations rather than the underlying substantive question about treatment provision. For example, Nadine explained:

[in mediation] you … spend the time looking at the sort of more practical stuff like, well, when will it [treatment withdrawal] happen? And how will it happen? And who will be there? And can it be in a hospice? Or, you know, other kind of elements of it.

Finally, and this was the least common outcome described to us, was where a ‘third way’ was agreed. Nadine explained this not as agreements which compromise the child’s best interests, but as certain factual scenarios where:

you get agreement that certain things will be tried in the future, but if they don’t work, then it’s palliative care … so kind of an example would be … a child that’s in hospital and not presently in intensive care on mechanical ventilation but is likely to relapse and need that at some point in the future. And you might get some kind of—I’ve had kind of really complex detailed orders that arrived effectively through a process of mediation, which say, “If the child deteriorates, then they will be admitted to intensive care, and they will receive mechanical ventilation but only for forty-eight hours, at which point, the doctor will review them, and if they’re not getting any better—” you know, it just sort of goes on and on and on. So there is sometimes scope for kind of… detailed kind of things like that.

These four potential outcomes in mediated cases were seen throughout the interviews and none of them, in our view, highlighted any evidence of compromise over the child’s best interests. It is noteworthy that there were no examples shared with us where the HCPs changed their position and agreed with the way forward proposed by family members at the mediation.

### A. Compromise

Several participants in our study explained that they thought mediation was about compromise and it was suggested, exclusively by HCPs, that the need to compromise can overshadow the need to prioritize the child’s best interests. This concern about mediation is fundamental; if mediation does lead to agreements which compromise the child’s best interests, then it is unlikely to be a suitable process for medical treatment dispute resolution even if it more effectively supports families. However, we found no evidence that mediated agreements undermined the child’s best interests. We based this assessment of compromise over best interests on the likely judicial interpretation of it. Notwithstanding criticism of the standard for its imprecision and uncertainty,^32^ we do know that courts are more likely to agree with the best interests outcome preferred by the public body.^33^ This suggests that any departure from the HCPs’ view on best interests is more likely to indicate compromise because we know courts are more likely to agree with HCPs.^34^ We also analysed the outcomes shared by our participants and their views on those outcomes and found that they did not subjectively view agreements as undermining the child’s best interests, despite their expressed concerns about the possibility of compromise.

It was suggested by some HCPs that high-profile cases such as *Charlie Gard* or *Archie Battersbee* have instilled a sense of caution among clinicians to the point many would be more willing to accede to the parents’ request, possibly more than they would feel comfortable doing, because they want to ‘avoid conflict or going to court’ (Yasmin, paediatrician). Yasmin believed that, because of these high-profile cases, clinicians are ‘more and more…conceding to compromising on the child’s best interest[s], really, to give parents what they want or what they’re asking for’. These comments were made more generally about clinical practice rather than specifically about mediation. However, Yasmin further expressed that in mediation, for a situation to escalate to the point of needing mediation, it would have to be because a doctor is:

super convinced that the best interests of the child won’t be met unless there’s agreement from the parents. So, it is kind of a bit of a sort of paradox really, isn’t it? Because then you would have to compromise on what you think is in the child’s best interests. But there might be tweaks that can be made that would feel, not comfortable, but kind of acceptable to you as a clinician, I suppose. (Yasmin, paediatrician)

Yasmin felt that a situation like mediation puts an onus on clinicians to be flexible and open to agreement, which could require clinicians to compromise. However, she also acknowledged that getting parental agreement on the way forward might also be in the child’s best interests, highlighting the complexity of the best interests judgement that HCPs engage in, which may not always be an unsatisfactory compromise by reaching an agreement with parents about a way forward. This latter view reflects some academic commentary on compromise, with Huxtable, for example, seeing the role of compromise in ethically challenging scenarios in a more positive light.^35^ Moreover, he links this idea of compromise to the many challenges in drawing boundaries around the best interests standard and identifying what it actually requires of clinicians and judges.^36^ Similar arguments have been made in the context of adults, with Kong et al. critiquing the difficulties faced in weighing incommensurate values when applying the best interests test under the Mental Capacity Act 2005, which can often obscure the underlying values engaged by decision-makers.^37^

Several other HCPs shared the sentiment that the clinical teams in disputes typically feel they do not have anything more to offer at mediation, implying that mediation would require compromise, which they are not in a position to do. One participant (Rowan, paediatric intensive care consultant) expressed that clinicians usually make many attempts to reach a resolution before initiating proceedings, and so he felt there is little more that mediation can achieve, again, because compromise is not on the table for them. Our data showed several clinicians echoing that they only end up in dispute once they are convinced their position would be in the child’s best interests, which means there is no room for negotiation and, in some cases, doing so would undermine best interests. This does not mean that HCPs do compromise in mediation; rather, they feel they cannot because it would not be in the child’s best interests, and so mediation is unlikely to be effective.

One participant (Caleb, paediatric intensive care consultant) felt that ‘the mediation process is on to a loser there from the outset’ because ‘the clinical teams aren’t left with anything to give’. This understanding of compromise in mediation is striking; it suggests that reaching a different outcome at mediation than that which was previously proposed by HCPs is inevitably a form of compromise. Whereas an HCP changing their previously held view at mediation could simply represent that they have changed their mind following a period of improved communication and understanding, or even that there may be several different ways of resolving the dispute, all of which could be within the zone of a child’s best interests. This understanding of mediation is widespread, and although compromise might be an element of mediated agreements in commercial disputes, in medical treatment disputes mediated agreements need not be the result of a ‘confidential carve-up borne of an unseemly horse trade’,^38^ rather a mechanism to facilitate finding agreement about a way forward. In contrast to several HCPs viewing mediation as a vehicle for compromise, non-HCP participants expressed fewer concerns about compromise, with one participant (Rachel, lawyer) explained she did not really see it as a real concern because she had not seen anything of this nature in the cases she had been involved in, although they were all cases where proceedings had been issued.

Despite general concerns about compromise, most participants remarked that they and the mediator always had the best interests question at the forefront of their mind. For example, it was noted that, in mediation, family members themselves reinforce that ‘the child is at the centre of everything’ (Tamara, consultant paediatrician), reminiscent of the paramountcy of the welfare of the child in the Children Act 1989.^39^ Further, mediators are adept at bringing the conversation back to the child and finding ways to emphasise the role of the child in the dispute to ensure their welfare is protected. For example, mediators would ask family members questions about the child, display pictures of them, and refocus the discussion around the child and their needs:

Ideally, I’d like to meet the child anyway, … generally with the parents, so because I think quite often there is a sense that … health professionals are not seeing the same child as parents are seeing, so… And also, it helps it helps me be authentic in my focus which is … all about the child, let’s keep the child at the centre. And the family know I’ve met them, and the health professionals know I’ve met them, even if they’re ventilated or [a] baby, or whatever. It’s always nice to meet them and meet them in person. (Abigail, Mediator)

… what helped was inviting all of the parties to tell me about P, and particularly the family who asked if they could show me photographs, which I was really happy about. I don’t always say, “Can you show me photographs?” Because that might feel intrusive. But it’s so helpful to me when people feel like they would like to. So that was the—you know, the closest route that I could get to understanding P. So understanding—not understanding P, but knowing a little bit about who P was. (Elizabeth, Mediator)

Most notably, when participants were asked if they thought any agreements reached in the mediations they had personally been involved in was in the child’s best interests, several remarked that they thought it was and were satisfied with the decision. This is an important finding because it highlights that those we spoke to generally perceived the child’s best interests to be met through mediation, even if outcomes were not in agreement with what individual clinicians may have entered the mediation initially hoping for. This is particularly so in the third type of outcome, where HCPs may agree to certain changes in approach such as around timing or location of treatment withdrawal. Reiterating the arguments above, this suggests that there may be more than one outcome (or outcomes) which can secure the best interests of the child.^40^ This may be controversial for HCPs who perceive there to be a single clinical answer to the best interests question, but best interests is a legal standard, not a clinical one, and its key strength arguably lies in its flexibility;^41^ it incorporates a range of factors, which will be fact-specific and go beyond clinical considerations.^42^ As Mr Justice Macdonald set out:^43^

The term ‘best interests’ is used in its widest sense, to include every kind of consideration capable of bearing on the decision, this will include, but is not limited to, medical, emotional, sensory and instinctive considerations.

This means that while the courts more often than not agree with HCPs on the substantive question around treatment or withdrawal, there may be scope for differences in approach to that process to meet the best interests of the child beyond their narrow clinical needs.

Despite there being a lack of evidence that best interests were compromised, we are conscious that we only interviewed one family member and one family supporter for this research. It is possible that family members’ perspectives on mediation and its outcomes might be different from mediators and HCPs, highlighting a limitation of this study. The one family member we did interview (Lola, patient’s mother) initially expressed some cynicism towards mediation, most likely borne out of the deep sense of distrust towards the clinicians due to previous encounters. Lola was involved in a dispute concerning the medical treatment of her newborn son, who was born with a rare mitochondrial disease and, within 5 days of his birth, was put on life support treatment. Because of the low survival rate, the clinical team had suggested that life support be withdrawn, but his parents did not agree to this, and so for several months the child remained in the hospital. During this time, tensions rose between the parents and the treating team. Not only were there ongoing discussions regarding the withdrawal of treatment, but the mother felt that ‘so many things went wrong’ during the child’s admission. In Lola’s interview, she explained that the clinical team suggested mediation, on the advice of the clinical ethics committee. Lola described feeling cynical about the mediator at their first meeting, expressing concern that she might be biased and be a tool of persuasion to get her to agree with the hospital. However, these fears did not materialize and, in fact, she described her experience with the mediator in very positive terms:

… Her [the mediator’s] approach, her calmness, her unbiasedness, because that was what I was looking for. You understand? Because my initial fear was whoever they were going to bring will not be truthful. Or will be one-sided … so that if the person is biased in a way, it … was playing on my mind. So hence why I was a bit not welcoming to [the mediator]. But when we started the meeting, I could tell she was not on any side. She was pretty straightforward. But, actually, that was the beginning of the whole turnaround. (Lola, patient’s mother)

Moreover, Lola expressed feeling comfortable with the decision reached in mediation, and, when asked, she said that she felt the decision was in her child’s best interests:

Interviewer: Okay. And did you think that the agreement reached, that care plan reached, did you feel that was in your son’s interests? Were you happy that it was the right thing for him?

Lola: Yes. Yes, it was. Yes … It was. It was. And I felt like that coming, the mediator being involved, was the best thing that could have happened to us in that moment. (Lola, patient’s mother)

Lola’s reflections, while only indicative of her own experience, show that mediation can support families in medical treatment disputes and does not have to lead to compromise in ways that undermine the child’s best interests; even where an individual changes their position in mediation, as Lola did by agreeing to withdrawal of treatment, that does not necessarily reflect a compromise. Instead, Lola saw it as securing the best thing for her and her family, and it enabled her to accept the withdrawal of treatment in ways that she had previously been unable to.^44^ Despite the many expressions of concern about compromise outlined above, there were very few examples where a child’s best interests had been obviously undermined by the mediation process or the outcome. For example, one participant (Rowan, paediatric intensive care consultant) explained:

the longer that things go on the more it’s difficult for people, the more it might feel empowering for families but actually isn’t it’s more, it’s empowering for people who sell newspapers for the very big cases I’m afraid. At the bottom of it there is a patient who is suffering, that’s the slight challenge here, very often the prolongation of things doesn’t really help the individual.

His interview provided a frank and reflective analysis of the real challenges in resolving these sorts of disputes and again hinted at concerns about compromise in these cases. However, he did not highlight any specific examples where mediation had been used by the family to, on his own account, undermine the child’s best interests. Instead, his reflections highlighted the problem of delay, and it is this issue to which we turn next.

### B. Delay

One concern over best interests is where there is a delay in the resolution of the dispute because the HCPs seek to resolve the matter by agreement, rather than through a court order. Such an approach has been criticized by the courts in cases concerning adults and children,^45^ with Mr Justice Hayden confirming, ‘There is no onus on the ICB or healthcare providers to broker an agreement between family members. … Mediation in these circumstances risks conflating the family’s views of best interests with the authentic views of P himself’.^46^ Of course, there are many differences between cases concerning adults and children, but the way in which delay has been treated by the courts has been strikingly similar.^47^ This could be delays because of trying to seek agreement, obtain a clearer idea of the person’s views (particularly for older children), secure the parents’ engagement, attempts to engage in formal, independent mediation, or even for intentional reasons to allow family members more time with their loved ones.

One of the primary examples of delay was from an interview with an HCP (Caleb, paediatric intensive care consultant) who recounted his experience of spending over 18 months trying to reach a decision regarding the withdrawal of treatment for a young child. In that case, the child, who was two and a half and was hospitalized as a result of severe birth asphyxia and hypoxic ischemic encephalopathy, had never left the hospital. The child had been on a ventilator since birth, was blind and deaf, and had extreme neurological injury with no demonstrable awareness of their environment, and it was the consensus of the treating team that the child derived no benefit or enjoyment from life. The child’s mother held strong religious views and, according to Caleb, believed that ‘God is going to make the child better’. Caleb recounted the process being ‘longer than typical’ because they ‘had [a] real problem getting an external second opinion from a neurologist’ and the Trust agreed to keep waiting. Throughout this time, the Trust refrained from issuing proceedings as they were trying to ‘check off’ mediation first, as Caleb described. This approach could be viewed as motivated by a desire to compromise, or at least be willing to engage with the family of the patient, an approach not dissimilar to reported judgements where attempts to mediate and the resulting delay have been criticized, despite possible benefits to delay from the family’s perspective.^48^ Moreover, in a case with similar facts but where the family agreed with the HCPs on the course of action, it would seem highly unlikely that the HCPs would instruct a second opinion, therefore suggesting this was done to appease the family. When they eventually engaged in mediation, Caleb recounted expecting it to be ‘futile’ due to the patient’s mother being ‘extreme in her behaviours and opinion’. He expressed the view that the family was not open to changing their position at mediation and acknowledged that the healthcare team, too, was unlikely to change their position due to the stage of the dispute at which they mediated:

And by that time, you know, everybody’s written their statements, which, because of the- the legal structure we have to work with it, are very black and white. And, you know, we don’t have anything to give at that stage because, you know, the only decision is … whether the treatment is in the child’s best interests or not, and everybody is completely entrenched at that point. Any compromising suggestion has been beaten out of people’s opinions by the time they get there, so the only time that- that we have something to give from the clinical side is at the outset in the early weeks and months. (Caleb, paediatric intensive care consultant)

Ultimately, the mediation did not resolve the case, nor did it appear to have any other benefits, with Caleb describing it as ‘a waste of everybody’s time’ and saying that it ‘wasn’t helpful’. It is worth noting that, even at the outset of the mediation, Caleb felt that ‘it was really clear that it was never gonna be helpful’, but the Trust still attempted mediation because they felt an expectation to do so, something we return to in the coercion section below.

Caleb’s case was his only direct personal experience of mediation, although he explained that his department had engaged in several mediations over the years in similar cases with varying degrees of success. The case highlights the challenge, and the concern, about mediation and best interests disputes; it was the delay in this case which negatively impacted upon on the child’s best interests, rather than mediation per se. Waiting over 18 months for an expert report and to mediate a ‘futile’ case is highly unlikely to be regarded as in the child’s best interests under the current legal framework, even taking into account the criticism of the subjectivity within best interests judgements. While this cannot be solely attributed to the mediation, there were aspects of the mediation attempt that were concerning. For example, if none of the parties were open to resolution at mediation, it may not have been the right step to take, particularly considering the delay that had already occurred due to the expert report. To echo the words of Justice Hayden, ‘the timescales of such mediation must be guided by those of the child/patient’.^49^ Caleb’s case study illustrates that many of the concerns we identified regarding mediation and best interests were not really concerns about compromising on best interests at all; rather they were concerns about the delay that mediation can cause which, in turn, risks (albeit not in all cases) undermining the best interests of the child. There was evidence from other cases that mediation can be used to prolong disputes. Rowan explained his experience of one mediation around 2014 where the mediators ‘came along, they talked, they charged money, the family didn’t find it useful, we didn’t find it useful.’ Nadine, an experienced lawyer and mediator, remarked that ‘it [mediation] definitely does prolong it [the case]’ (Nadine, lawyer) and another participant (Jack, paediatric intensive care consultant) described a case in which mediation prolonged the dispute by another 2 months before they finally went to court to get a decision to stop treatment. Those 2 months undoubtedly extended the child’s life, but whether that was a positive effect or not was questionable:

… arguably it will have extended her life somewhat, because…so she was going through these cycles of stopping breathing, ambulance into hospital, plugged onto a ventilator. And after a week or two on the ventilator, she’d start to breathe again and then would go home … and the mediation process took, I mean the actual meetings took about 2-3 weeks I think, but it took about a month before that to find a date and coordinate it all, because obviously everyone had to be free, so I think the whole thing probably took two months in the end. So we had two months… And that cycle, that cycle was continuing through all of that. So… in the sense that it took two months longer before we then went to court and, then got the court order to stop, it, you can argue it added two months of life to her, and the parents valued the time they had at home with her.

Interviewer: So not necessarily a detrimental impact on her.

Jack: It depends on how you view the process of coming into intensive care and, and whether you have…. I, I didn’t feel she had any awareness or experience of it, of life. But, if she did, it was probably worse, that… was probably a bad thing for her. (Jack, paediatric intensive care consultant)

In this case, the participant felt that mediation was futile because it was clear early on that there was not much scope for common ground between the parties and, because of the parties’ entrenched positions, it was unlikely that they were going to reach an agreement. The same participant also recounted another case where the hospital trust had attempted mediation with a family who wanted them to continue treating despite the hospital trust wanting to withdraw treatment. In that case, it was explained that the mediation was unsuccessful and almost destroyed the relationship with the family and, upon finally taking the matter to Court, the hospital trust was criticized by the judge for taking so long to issue proceedings, highlighting the central challenge to mediating best interests disputes is delay, rather than agreements which undermine the child’s best interests.

If, as our research suggests, mediation can cause delay, then perceptions of the utility of mediation are likely to be impacted by whether delay is perceived to undermine the child’s best interests. There may not be one answer to that question; it may depend on the individual facts of the case and one’s view on the weighing up of life versus suffering,^50^ but it does, at least, seem likely that mediation adds an additional layer of delay, which could be seen to negatively impact the child. However, delay is likely to be viewed by parents, particularly those in conflict with HCPs, as a positive aspect for their child. It can give them more time with their child than they otherwise might have had and, in some instances, lead to partial agreements following the mediation which would not otherwise have occurred if the family had agreed with the HCPs or court proceedings had been immediately sought. For example, in the case of *GOSH v MX & FX & X (Re X)*,^51^ following mediation, the parents agreed that it was not in the child’s best interests to be subjected to various interventions, although the parents still disputed the issue regarding the provision of oxygen and readmission to the PICU.^52^ Proceedings had been adjourned in order for parties to attempt mediation and, while there may be concern that this would cause a delay in reaching a decision, it appears that mediation helped narrow the issues in dispute in this case as ‘the meetings allowed for an exchange of information about X’s condition and her parents were able to voice their concerns’.^53^ Moreover, this decision was partially in favour of the parents, which, as one of the authors has argued elsewhere, is an unusual outcome in children’s medical treatment disputes.^54^

Our research also confirmed that not all medical treatment disputes that reach mediation concern withdrawal of treatment and some may in fact concern the administration of treatment. In such cases, especially if the treatment is lifesaving, a delay in reaching a decision may be even more detrimental, in which case the parties may not be able to afford the time it takes to mediate. One HCP (Rowan, paediatric intensive care consultant) noted that actually ‘quite a few’ of the cases that go to court are concerning ‘treatment orders to get a child treatment that the parents have declined, that’s life sustaining’ as opposed to withdrawal orders to discontinue treatment. Another participant (Sonny, consultant paediatrician) described a case in which the dispute concerned a blood transfusion that the child needed but the parents refused on religious grounds. This highlights that delay caused by mediation could also be problematic in cases where the parents, rather than HCPs, refuse medical treatment for their child.

It is important to consider the impact of delay on the child’s best interests when contemplating mediation in any dispute. We suggest that before mediation is recommended, there is a need to weigh up the high risk that mediation will cause some delay to final resolution and the arguably lower likelihood of the potential benefits of reaching (partial) agreement in ways that both family members and HCPs can support and feel satisfied with. The precise balance of these factors will vary depending on the facts, but it indicates to us the need to at least have some potential for benefits to accrue for the risk of delay to be worthwhile. Specifically, we consider that, as a minimum, the parties must show some openness to mediation and ought not feel coerced into the process (something discussed further below). We did ask mediators if there were any cases they would refuse to mediate and several responded indicating that parties ought to be open to mediation. For example, one participant explained:

I have refused to mediate a case on the basis that I didn’t feel we had full buy in from all the parties, I felt genuine resistance and … in those instances sometimes I signposted either to other mediators or … recommended maybe them going back, you know maybe taking legal advice or even therapeutic advice. And then I have ended mediation where I felt … that the parties weren’t able to engage in a successful manner and where there were safeguarding issues that I felt weren’t being addressed. (Elizabeth, mediator)

This indicates that the mediator may be best placed to identify openness to mediation in pre-meetings or even during a full mediation session, but there is a potential conflict of interest in those circumstances if the mediator has already been instructed to mediate the dispute. Moreover, although several mediators noted they would refuse to mediate in these circumstances, some also noted they had never refused to mediate cases before. For example, Abigail (mediator) was asked whether there would be any cases which she would refuse to mediate and she initially answered ‘I think I see the possibility of mediation … it’s a really valuable thing. So I haven’t declined anything. I guess what I’m trying to say is, I find it hard to think of something I would decline’ but she went on to say ‘there is one I think I would refuse … It’s something where the person says “I only want this and nothing’s gonna change my mind”, then I might say ‘I’m not… I don’t think I’m not sure there is territory for mediation”’.

In this section we have shown that there was no evidence from our research that agreements reached in mediation result in compromise which undermines the child’s best interests, either judged subjectively by our research participants or judged against how a court would likely interpret the best interests issue. In fact, the primary issue impacting on best interests was mediation’s role in causing delay in resolution because it does not lead to an agreed way forward. This means that where there is potential for delay, this needs to be weighed against the potential benefits of the mediation process.

### C. Coercion, pressure, and power imbalances

Mediation, being non-binding, does not have the authority of a court order and so is not coercive in that sense because HCPs and family members can choose to walk away without agreement. Nevertheless, there were concerns about mediation’s coercive effect because power imbalances can entrench coercive practices and make parties feel pressured into participating or agreeing to actions at mediation. This could occur in several different ways, but we focus here on what we call ‘outcome coercion’ of family members by HCPs and ‘process coercion’ through parties feeling obliged or even forced to participate in a mediation, which may also prolong the dispute.

#### 1. Outcome coercion

One concern is that mediation can be used by HCPs to coerce family members into an outcome with which they do not agree and/or may not be in the child’s best interests. This is linked to the well-established power imbalances between the parties in healthcare.^55^ A few participants highlighted the inherent power imbalance in medical treatment disputes in that ‘the power does lie, objectively… with the clinician, profession or the Trust’ (Ed, mediator), although a smaller number of HCPs and one chaplain felt the balance had now shifted, explaining ‘I think there’s a really sort of tired narrative about doctors being paternalistic and saying what they think … should happen and all the rest of it. And I just feel like it’s completely the other way round now’ (Yasmin, paediatrician).

Some mediators and HCPs described parents struggling with the decision that was reached at mediation, suggesting they reached agreements they may not have been comfortable with or that mediation may not always be a supportive process for families coming to terms with the loss of their child. For example, one participant (Abigail, mediator) spoke of a parent involved in a mediation: ‘… she was and she is still wrestling with that now’. Having spoken with the parent years after the mediation, the participant learned that, despite agreeing to the final decision to have their child extubated, the mother still struggled with this decision in the years to follow. This participant recounted how the mother told her that there were times where she was in the hospital and she was wrestling with whether to raise an issue but hesitated out of fear that she would upset the professionals. The participant acknowledged that the mother appeared settled in the decision at the time (‘both parties were settled in it’) but, in hindsight, the mediator expressed the opinion that they would have liked to have spent more time exploring these past issues so that the mother could come to terms with the decision better and make her peace with the situation for the long term without any hint of coercion in her reflections.

The family member we interviewed (Lola, patient’s mother), despite initially saying she would not agree with the HCPs’ recommended treatment, did agree with the proposed course of action of life support being withdrawn. Importantly, her recollection does not reference any outcome coercion. Instead, she credits the approach of the mediator as being a decisive factor in her agreeing to withdraw treatment:

So, initially, when she came, she was asking us the way forward. If we felt like—we said—I told her—I said, “We’re not going to agree. We’re going to, like, go to court.” But still, she still said, “Okay,” but she was going to call for a meeting. A final meeting. So when we came for that meeting, she said some things about—from our own views, and she said the medical personnel, their own views as well. So when she finished talking… because of her—I could tell because of her approach and the way she handled the situation, we agreed that the life support machine would be removed … We agreed. And that was when I told her—I said, “You know what? Your involvement and your approach towards these things … your sincerity, your transparency …” You know, the compassion as well in her approach. (Lola, patient’s mother)

As mentioned above, Lola said that she felt comfortable with the decision and reflected positively on the mediation, saying that it ‘saved’ her ‘personally, as a mother, from mental health [problems]’ and she even stated that she wished that the ‘the mediators were involved earlier’ in their journey because it ‘saved’ them from ‘going to court’. Our data suggested that although perceptions of coercion and power imbalances exist, the actual experience of coercion through mediation may well be overblown.

Despite Caleb’s earlier statement that they feel compelled to mediate, there are no court rules or guidance that require HCPs to mediate. In fact, a court is more likely to criticize delay than a failure to mediate, and evidence shows that if the hospital trust applies to the court, then the court order is more likely to be in agreement with them than in agreement with the family’s wishes.^56^ Therefore, HCPs may mediate because they want to seek agreement with the family rather than coerce them, as applying to court is highly likely to result in an outcome in favour of the HCPs anyway. A similar point is made by one of our legal professional participants (Nadine, lawyer), who expressed the view that mediation is used more as a tool to simply help the family ‘come to terms’ with the medical professionals’ decision and ‘understand the medical evidence and why the doctors are saying’ what they are saying:

Interviewer: So it sounds as if you’re saying that mediation, in this sphere, it’s more a tool of persuasion by the Trust for the patient or their family rather than a joint decision-making process. Would that be correct?’

Nadine: Yeah. That’s definitely how it feels to me. Sometimes there is scope for an actual third way. So, for example, you might end up with a case where you get agreement that certain things will be tried in the future, but if they don’t work, then it’s palliative care … So there is sometimes scope for kind of… detailed kind of things like that. But… in a lot of the cases that come to the court … a lot of them are about withdrawal of treatment from patients in intensive care. And my own view is that there isn’t actually a lot of scope to mediate those cases because staying in intensive care indefinitely is not an option that any doctor in this country will advise is in a patient’s best interest. And so, it doesn’t really matter. You know, you can get your second opinion from wherever you like. If it’s from England and Wales, it’s going to say the same thing as the treating doctors. (Nadine, lawyer)

These quotes highlight that in the mediation of medical treatment disputes, there is value in persuasion, rather than coercion, to reach an agreement when conclusive resolution is impossible. Where both parties’ interests cannot be met—for example, where what one party wants cannot be countenanced by the other and might jeopardize the child’s best interests—mediation can act as a vehicle for improving understanding, communication, and acceptance on the part of family members. It is therefore to be viewed as a therapeutic process for family members, something which perhaps distinguishes medical treatment mediation from other areas of mediation practice. While persuasion of this kind risks being seen as ‘getting the family on side’ with the HCPs’ position, in reality, the balance of power is not solely with the HCPs, as power in a mediation context comes in many forms. This is reinforced by perceptions from another participant (Marcus, chaplain), who noted he believes that the power balance between clinicians and families has shifted:

And I think for clinicians, one of the things I notice a change in the… years I was at the hospital and … things have changed for clinicians, so you know I came in probably at the end of it being as the whole internet explosion was happening in a way, but a lot of people would come, and what the clinician said was what the clinician said. Now there is much more the clinician will say to you … this is our clinical point of view and then the … parents will get a pile of documents out of their bag and say but you know in Kazakhstan, or in wherever it is, there’s this doctor who’s doing this treatment, why aren’t you considering that? So that whole power balance between the clinician and families has changed. (Marcus, chaplain)

The earlier concerns about the inherent power imbalance between HCPs and families, in which the lack of knowledge creates an unlevel playing field in mediation, is starting to level out as access to information and support for families grows. Taken together, it seems that mediation can be an effective way of sharing information to improve understanding of the respective positions and experiences of the parties and thus to improve communication, which can only be positive for supporting families in conflict of this nature. Resolution may not be the appropriate measure of success for medical treatment disputes mediation, where parties can, instead, use the process to achieve a greater level of acceptance of the decision based on improved communication and understanding.^57^

#### 2. Process coercion

Finally, we identified some evidence of process coercion, where HCPs felt obliged to attempt mediation even where they felt it was futile or unsuitable for the case. Most participants expressed this pressure coming from the courts, but one participant (Tamara, consultant paediatrician) felt this pressure from the mediator too. This issue was identified in several of the HCP interviews and was most clearly articulated by one interviewee (Caleb, paediatric intensive care consultant) who repeatedly explained that the reason his department mediates their disputes is because the courts have told them to:

We’re in the position today where clinical teams are expected to have gone through a mediation process by the time they end up in court. But I think … it’s still at the moment more of a tick box exercise at the end of that pathway. (Caleb, paediatric intensive care consultant)

He was asked where that expectation to mediate had come from and he explained ‘it’s from the courts … I mean, that’s what’s driven it.’ Rowan similarly explained he feels there is pressure to mediate from the courts:

But then they were offered it in court and our legal team refused and said this is ridiculous, it’s just a stalling tactic so the judge went well you must have mediation used from now on, that’s one of the reasons it came in, erm, and so it became a thing where you had to have mediation whereas our legal team had said well there’s no point, you can’t mediate, this is not something that will work in mediation, and I agree with them. It would never have been, it would never have worked because of some things I can’t particularly share. Erm, however, it became well you must try everything before you approach the courts now, so then mediation ended up being de rigeur for the next, probably 5 or 6 years, with everyone around the country, and yet, it didn’t really make a difference to any of the cases that were in court. (Rowan, paediatric intensive care consultant)

This type of process coercion felt by HCPs was somewhat surprising, given recent judgements noted earlier, some of which have criticized the use of mediation, particularly where it causes delay. However, this also highlights the difficulties with legal literacy and the nuance with which the courts have treated mediation. Moreover, while mediation has been discussed in a small number of cases, it is not widely acknowledged by the courts, most likely because of its relatively limited uptake. While there has been some judicial support for mediation’s use,^58^ as noted earlier, recent case law has emphasized a more careful approach and we may yet see further engagement with mediation’s use which may or may not be positive. Perhaps, though, this perceived changing of judicial position is what Rowan was alluding to above when he described mediation’s emergence in recent years. In *Newcastle Upon Tyne Hospitals NHS Foundation Trust v H*, Mr Justice Hayden noted some of the perceived benefits of mediation,^59^ but he also went on to state that:

the timescales of such mediation must be guided by those of the child/patient, taking care to identify the point where they might diverge from the timescales of the parents or family. When there is a plan to discontinue treatment, on the basis that it is both futile and burdensome, it is ordinarily likely to be in the best interests of a child for there to be agreement as to the way forward, on the part of all concerned. That is most likely to protect the child’s dignity at the end of life. The lodestar, however, must always be the needs of the child.

These important comments highlight the challenge in mediating best interests disputes: on the one hand, mediation is perceived to have benefits for families in improving communication and understanding between the parties. On the other hand, the guiding principle in these cases ought always to be the best interests of the child such that where mediation creates delay, this must be considered through the prism of best interests and not the interests of the other parties involved. If HCPs feel pressure to mediate, even in cases where they do not feel it is appropriate, then this could have negative consequences for the best interests of the child. This reminds us that mediation may not be suitable for all cases. Here we have suggested that cases where the parties feel coerced into participation in the mediation process are not appropriate for mediation. Further research is needed to carefully pin down which cases might be most suited to mediation and the features of those cases.^60^

## V. CONCLUSION

This article has engaged with the question of whether mediating medical treatment disputes can be helpful in securing the best interests of the child outside of court. In seeking to answer this, we have analysed data from our 30 interviews and highlighted three themes that arise which engage with the best interests question: compromise, delay, and coercion. We have very little evidence of compromise agreements in mediated best interests disputes; instead, we see that participants are unwilling to compromise if they perceive that to undermine the child’s best interests but also that resolutions can be reached at mediation which parties feel provides a way forward. We have, however, found evidence that mediation causes delay in best interests disputes. We have noted that whether this undermines best interests is a matter of dispute, because it will depend on whether delay is perceived to be in the child’s interests. Different parties to the dispute are likely to see this issue differently, and even HCPs who may not support delay note that, in some instances of shorter delay, for example, it may not be problematic for the child. Indeed, some participants suggested there might be benefits resulting from delay, such as improved communication and relationships, that are in the child’s best interests and support the family. There is disagreement within the literature about the meaning of best interests and the extent to which it is a subjective concept or guided by a clear legal principle. While we have not directly engaged in that debate here, it will be an important consideration for those choosing to mediate because of the evidence that mediation can cause delay. Finally, we have considered the role of coercion. We have shown that there is little evidence of coercion of family members by HCPs, albeit we note that our evidence from family members is limited. We do see some evidence of process coercion, particularly of HCPs, some of whom appear to feel mediation is required by the courts. Overall, however, the evidence regarding mediation’s use in best interests disputes is positive; we saw few examples of the child’s best interests being undermined and some situations in which the use of mediation led to improved outcomes.

## Footnotes

1
*Great Ormond Street Hospital v Yates & Ors* [2017] EWHC 1909 (Fam) [para 20] and also discussed at *Great Ormond Street Hospital v Yates & Ors* [2017] EHWC 972 (Fam) [para 130]; J Lindsey, M Doyle and K Wazynska-Finck, ‘Navigating Conflict: The Role of Mediation in Healthcare Disputes’ (2023) 19 *Clinical Ethics* 1, 26; J Lindsey, M Doyle and K Wazynska-Finck ‘Securing Therapeutic Justice Through Mediation: The Challenge of Medical Treatment Disputes’ (2025) 45 *Legal Studies*, 40.

2 Since Charlie’s death, his parents have advocated for legal reform, known as Charlie’s Law. The reform is advocated for by the Charlie Gard Foundation: <https://thecharliegardfoundation.org/about/what-is-charlies-law/> accessed 23 May 2023. One of its key elements is to make mediation available whenever the difference of opinion cannot be resolved informally. Another important element is to restrict court involvement to cases where there is a risk of significant harm to the child. The proposal was introduced as an Amendment to the Health and Care Act 2022 by Baroness Finlay of Llandaff but has not been incorporated into law at the time of writing.

3
*GOSH v Gard*.

4
*Tafida Raqeeb v Barts NHS Foundation Trust & Ors* [2019] EWHC 2531 (Admin) and [2019] EWHC 2530 (Fam); *Barts Health NHS Trust v Dance & Ors* [2022] EWHC 1435 (Fam); *Kings College Hospital NHS Foundation Trust v Thomas &Ors* [2018] EWHC 127 Fam; *GOSH v Gard*; J Lindsey, D Schuberg and J Browning, ‘Medical Treatment Disputes and Children: An Empirical Analysis of Sixteen Years of Reported Judgments in England and Wales’ (2025) 46 *Journal of Social Welfare and Family Law* 1.

5 ibid.

6 Lindsey, Doyle and Wazynska-Finck, ‘Securing Therapeutic Justice’ (n 1)

7
*Re Y (Mental Patient: Bone Marrow Donation)* [1996] 2 FLR 787; *Tafida Raqeeb v Barts NHS Foundation Trust & Ors* [2019] EWHC 2531 (Admin); *AN NHS Foundation Trust v MC* [2020] EWCOP 33; *Manchester University NHS Foundation Trust v Fixsler & Ors* [2021] EWHC 1426 (Fam).

8 C Auckland, I Goold and J Herring (eds), *Parental Rights, Best Interests and Significant Harms: Medical Decision-Making on Behalf of Children Post-Great Ormond Street Hospital v Yates* (Hart 2016); C Auckland and I Goold ‘Re-evaluating ‘best interests’ in the Wake of *Raqeeb v Barts NHS Foundation Trust & Anors*’ [2019] EWHC 2531 (Admin)’ ‘(2020) 83 *Modern Law Review*, 1328; C Auckland, I Goold and J Herring (eds), *Medical Decision-Making on Behalf of Young Children: A Comparative Perspective* (Hart 2020); J Brierley, J Linthicum and A Petros ‘Should Religious Beliefs Be Allowed to Stonewall a Secular Approach to Withdrawing and Withholding Treatment in Children?’ (2013) 39 *J Med Ethics*, 573; TM Pope ‘Guest Editorial: Charlie Gard’s Five Months in Court: Better Dispute Resolution Mechanisms for Medical Futility Disputes’ (2018) 44 *Journal of Medical Ethics*, 436.

9 ibid.

10 See Lindsey, Doyle and Wazynska-Finck ‘Navigating Conflict’ (n 1); ‘Disagreements in the Care of Critically Ill Children’ (Nuffield Council on Bioethics 2023) <NCOB-web-version-independent-review-disagreements-in-the-care-of-critically-ill-children-september-2023.pdf> accessed 7 May 2025.

11 ibid; V Neefjes ‘Can Mediation Avoid Litigation in Conflicts About Medical Treatment for Children? An Analysis of Previous Litigation in England and Wales’ (2023) 108 Arch Dis Child 715.

12 Other types of mediation include transformative and evaluative, for further analysis of mediation see C Menkel-Meadow (ed), *Mediation: Theory, Policy and Practice* (2nd edn, Routledge 2018).

13 H Pincock ‘Does Mediation Make us Better? Exploring the Capacity-Building Potential of Community Mediation’ (2013) 31 *Conflict Resolution Quarterly*, 3, 13.

14 For further discussion in a similar context of medical treatment disputes concerning adults, see J Lindsey, *Reimagining the Court of Protection: Access to Justice in Mental Capacity Law* (Cambridge University Press 2022).

15 Our research showed that only three participants had been involved in formal mediations concerning children who were aged over five, with most participants describing mediation processes concerning younger children.

16 Lindsey, Doyle and Wazynska-Finck, ‘Navigating Conflict’ (n 1).

17 J Lindsey, G Francis and M Doyle, *Mediation of Medical Treatment Disputes: A Therapeutic Justice Model* (University of Reading 2025).

18 Lindsey, Doyle and Wazynska-Finck, ‘Securing Therapeutic Justice’ (n 1).

19 Lindsey (n 14); J Lindsey and C Danbury, ‘Mediating Disputes Under the Mental Capacity Act 2005: Relationships, Participation, and Best Interests’ (2024) 32 *Medical Law* Review 336.

20 Lindsey, Doyle and Wazynska-Finck, ‘Securing Therapeutic Justice’ (n 1).

21 Lindsey, Doyle and Wazynska-Fink, ‘Navigating Conflict’ (n 1); ‘Disagreements in the Care of Critically Ill Children’ (Nuffield Council on Bioethics 2023) <NCOB-web-version-independent-review-disagreements-in-the-care-of-critically-ill-children-september-2023.pdf> accessed 7 May 2025.

22 A Arstein-Kerslake and J Black ‘Right to Legal Capacity in Therapeutic Jurisprudence: Insights From Critical Disability Theory and the Convention on the Rights of Persons with Aisabilities’ (2020) 68 *International Journal of Law and Psychiatry*, 101535.

23 H Genn ‘Civil Mediation: A Measured Approach?’ (2010) 32 *Journal of Social Welfare and Family Law*, 195; R Hunter ‘Inducing Demand for Family Mediation—Before and After LASPO’ (2017) 39 Journal of Social Welfare and Family Law 2, 189; B Clark, Mandatory Mediation in England and Wales? A Short Note on the Civil Justice Council Report, “Compulsory ADR”. *The Australian Dispute Resolution Research Network Blog* 2021.

24 RA Peeples, S Reynolds and CT Harris ‘It’s the Conflict, Stupid: An Empirical Study of Factors that Inhibit Successful Mediation in High-Conflict Custody Cases’ (2008) 43 *Wake Forest Law Review*, 505; D Bagshaw and others ‘Elder Mediation and the Financial Abuse of Older People by a Family Member’ (2015) 32 *Conflict Resolution Quarterly*, 443.

25 Issues such as power imbalances between the parties, closedness to mediation and the role and skill of the mediator are not addressed here, but for further analysis see Lindsey, Francis and Doyle, (n 17).

26 The number exceeds the total number of participants as some participants had experience across adult and children’s cases.

27 The limited information we do have can be seen from analysis of reported case law, see Lindsey, Schuberg and Browning (n 4). That analysis identified 14 cases in a 16-year period which refer to mediation in the judgement and eight cases which appear to have been mediated.

28 As well as challenges in recruiting family members we also experienced difficulties in accessing mediations to observe. Three mediations were observed regarding adult health and care cases, this analysis will be published elsewhere. The reasons for the challenges in observing mediations concerning children are set out in Lindsey, Francis and Doyle (n 17).

29 See Lindsey, Francis and Doyle (n 17).

30 ibid; see J Lindsey, ‘Mediation as Therapeutic Resolution for Conflicts About Patient Health and Care’ (manuscript in preparation).

31 This is also discussed elsewhere in the context of mediation where family members held religious beliefs, see J Lindsey, ‘Mediating Religious Disputes About Children’s Medical Treatment: A Qualitative Study’ (forthcoming).

32 R Huxtable. *Law, Ethics and Compromise at the Limits of Life: To Treat or Not to Treat?* (Routledge, 2013).

33 Lindsey, Schuberg and Browning (n 4).

34 ibid.

35 Huxtable (n 32).

36 ibid, generally but specifically see chapter 4.

37 C Kong and others. ‘An Aide Memoire for a Balancing Act? Critiquing the ‘Balance Sheet’ Approach to Best Interests Decision-Making’ (2020) 28 *Medical Law Review* 753.

38 M Supperstone and others, ‘ADR and Public Law’ (2006) *Public Law* 299, 312–3.

39 I am grateful to the reviewer for this observation.

40 I am grateful to the reviewer for highlighting this point and for further discussion about the meaning of best interests see C Auckland, I Goold and J Herring (n 8); Huxtable (n 32); J Lindsey, ‘Best Interests and Relationality in Reproductive Healthcare’, in Davey and Lindsey (eds), *Grandparents and the Law: Rights and Relationships* (Bloomsbury Publishing 2023).

41 For discussion see Huxtable (n 32), in particular 85–90.

42
*Manchester University NHS Foundation Trust v Fixsler & Ors* [2021] EWHC 1426 (Fam) at [57].

43 ibid.

44 We acknowledge that some family members would not see this outcome as in the best interests of their child. However, we base our analysis on the strong likelihood that a court would find this decision was in the child’s best interests, see Lindsey, Schuberg and Browning (n 4).

45
*Newcastle Upon Tyne Hospitals NHS Foundation Trust v H* [2022] EWFC 14.

46
*NHS South East London Integrated Care Board v JP & Ors* [2025] EWCOP 8, para 34.

47 See eg the criticisms of delay in *Dean Gregory v Nottingham University Hospitals NHS Foundation Trust & Ors* [2023] EWCA Civ 1324.

48 ibid.

49
*Newcastle Upon Tyne Hospitals v H.*

50 Auckland, Herring and Goold, and Auckland and Goold (n 8).

51
*GOSH v MX & FX & X* [2020] EWHC 1958 (Fam).

52 Lindsey, Doyle and Wazynska-Finck, ‘Securing Therapeutic Justice’ (n 1), 15–16.

53
*GOSH v MX* (n 51), para 2.

54 Lindsey, Schuberg and Browning (n 4).

55 M Brazier and J Miola ‘Bye Bye Bolam: A Medical Litigation Revolution?’ (2000) 8 *Medical Law Review*, 85; C Foster and J Miola ‘Who’s in Charge? The Relationship Between Medical Law, Medical Ethics, and Medical Morality?’ (2015) 23 *Medical Law Review*, 505; P Case ‘Dangerous Liaisons? Psychiatry and Law in the Court of Protection—Expert Discourses of ‘Insight’ (and ‘Compliance’)’ (2016) 24 *Medical Law Review*, 360.

56 Lindsey, Schuberg and Browning (n 4).

57 Something reinforced by the procedural justice literature that where people are more involved in the process of resolution, they are more accepting of it, see TR Tyler ‘Procedural Justice, Legitimacy, and the Effective Rule of Law’ (2003) 30 *Crime and Justice*, 283; T Tyler, ‘Procedural Justice’ in A Sarat (ed), *The Blackwell Companion to Law and Society* (Blackwell, 2004); S Halliday and P Schmidt, ‘Tom Tyler and Why People Obey the Law’ in *Conducting Law and Society Research: Reflections on Methods and Practices* (Cambrigde University Press 2009); S Paul and others ‘Perceptions of Key Stakeholders on Procedural Justice in the Consent and Capacity Board of Ontario’s Hearings’ (2020) 68 *International Journal of Law and Psychiatry*, 1.

58
*GOSH v Gard*; *GOSH v MX*.

59
*Newcastle Upon Tyne Hospitals v H*, para 25.

60 For further discussion of some of these issues see Lindsey, Francis and Doyle (n 17); Lindsey (n 31); Lindsey (n 30); Neefjes (n 11).

